# Heat stress promotes GTBP-1 association with perinuclear P-bodies in the *C. elegans* germline

**DOI:** 10.1371/journal.pgen.1012336

**Published:** 2026-10-07

**Authors:** Diya Zang, Yifan Jing, Xinya Huang, Yan Kuang, Jiewei Cheng, Wenkai Wang, Demin Xu, Chengming Zhu, Di Chen, Zhongying Zhao, Xuezhu Feng, Shouhong Guang

**Affiliations:** 1 Department of Obstetrics and Gynecology, The First Affiliated Hospital of USTC, The USTC RNA Institute, Ministry of Education Key Laboratory for Membraneless Organelles & Cellular Dynamics, Hefei National Research Center for Physical Sciences at the Microscale, Center for Advanced Interdisciplinary Science and Biomedicine of IHM, School of Life Sciences, Division of Life Sciences and Medicine, University of Science and Technology of China, Hefei, China; 2 School of Basic Medical Sciences, Anhui Medical University, Hefei, China; 3 Zhejiang University-University of Edinburgh Institute, Zhejiang University School of Medicine, Haining, China; 4 Department of Biology, Hong Kong Baptist University, Hong Kong SAR, China; Temasek Life Sciences Laboratory Ltd, SINGAPORE

## Abstract

Stress granules (SGs) are dynamic ribonucleoprotein condensates that enable cells to adapt to environmental stress, yet their functions and regulation in the germline remain incompletely understood. Here, we characterize GTBP-1, the *Caenorhabditis elegans* homolog of human G3BP1, and investigate its role in heat stress responses and reproductive homeostasis. *gtbp-1* mutants exhibit temperature-dependent reproductive phenotypes. Under normal growth conditions, GTBP-1 is diffusely distributed in the cytoplasm, whereas heat stress induces the formation of prominent GTBP-1 granules in the germline. Domain analysis revealed that the NTF2 domain is required for heat stress-induced GTBP-1 enrichment in granules, while the IDR, RRM, and RGG domains contribute to GTBP-1 granule dynamics. Among the known germ granule compartments, GTBP-1 granules showed the strongest association with P-bodies, and disruption of P-body components markedly reduces GTBP-1 enrichment. RNAi-based candidate screening identified PQN-59, LAF-1, and SMO-1 as regulators of GTBP-1 enrichment, while a forward genetic screen revealed that loss of the mTOR effector RSKS-1 suppresses the fertility defect of *gtbp-1* mutants and alters heat stress-induced GTBP-1 enrichment. Together, these findings identify a role for GTBP-1 in temperature-dependent reproductive homeostasis and reveal an association between heat stress-induced GTBP-1 granules and P-bodies in *C. elegans*.

## Author summary

Reproduction is particularly vulnerable to environmental stress, yet germ cells must remain functional to ensure fertility. How germ cells protect themselves under stressful conditions is still not well understood. In this study, we used the nematode *Caenorhabditis elegans* to examine how germ cells respond to heat stress, focusing on GTBP-1, a conserved protein involved in stress granule formation. We found that GTBP-1 has temperature-dependent effects on reproduction: it restrains reproduction under normal conditions but is required for reproductive fitness under heat stress. When animals are exposed to elevated temperature, GTBP-1 rapidly forms granules in germ cells. These granules show their strongest association with P-bodies, another type of RNA-containing structure, highlighting a close spatial relationship between these two RNA-rich compartments. We also found that this process is regulated by factors involved in RNA regulation, protein modification, and mTOR-dependent signaling. Together, our findings show how germ cells reorganize RNA–protein assemblies in response to heat stress and provide new insight into the regulation of reproductive homeostasis under changing environmental conditions.

## Introduction

Organisms frequently encounter diverse environmental stresses, including temperature fluctuations, oxidative stress, and nutrient deprivation. To preserve cellular homeostasis, cells have evolved rapid and reversible response mechanisms, among which stress granules (SGs) represent one of the most critical stress-induced subcellular structures [1,2]. Stress granules are primarily composed of translationally stalled mRNAs and associated proteins, and their assembly is mediated by multivalent protein–protein and protein–RNA interactions that promote liquid-liquid phase separation (LLPS) [3,4]. By transiently sequestering mRNAs, stress granules modulate their translation, stability, and degradation, thereby dynamically regulating gene expression at the post-transcriptional level [5–7]. Notably, germ cells and early embryos are particularly vulnerable to environmental stresses, including heat stress, and the fidelity of reproductive processes relies heavily on precise intracellular homeostasis [8–10].

The formation of stress granules is orchestrated by a complex network of protein-protein interactions. Beyond the classic translation initiation factor eIF2, several nucleating factors, including Ras GTPase-activating protein-binding protein 1 and 2 (G3BP1/2), ubiquitin-associated protein 2-like (UBAP2L), and T-cell-restricted intracellular antigen-1 (TIA-1), have been demonstrated to be crucial for stress granule assembly [11–13]. Mechanistic studies indicate that G3BP1/2 drive stress granule formation through RNA-induced conformational changes and self-aggregation, a process that depends on the synergistic interplay between the NTF2L domain and RNA-binding domain (RBD) [14–16]. During heat shock, the subsequent disassembly of stress granules is mediated by ubiquitination of G3BP1 [17], underscoring its central role in the dynamic remodeling of stress granules. In *C. elegans*, GTBP-1, the homolog of human G3BP1, has been implicated in stress granule assembly and stress responses, while PQN-59 and TIAR-1 have been implicated in germline stress granules [18–20], providing an ideal model for studying the dynamic regulation of stress granules and their connection to reproductive function. Nevertheless, it remains unclear how GTBP-1 contributes to germline reproductive homeostasis under temperature stress and how its distribution and granule association are regulated by environmental conditions.

Germ granules are RNA-enriched condensates typically localized to the perinuclear region of germ cells, involved in RNA homeostasis and post-transcriptional regulation, and are crucial for germline development and reproductive homeostasis [21–23]. In *C. elegans*, germ granules comprise multiple substructures, including P granules, Z granules, Mutator foci, SIMR foci, D granules, E granules and P-bodies [22,24–26]. In yeast and mammalian cells, stress granules and P-bodies exhibit partial spatial overlap and undergo dynamic messenger ribonucleoprotein (mRNP) exchange under stress conditions, with some components being targeted for autophagic degradation [27–29]. Recent studies have shown that heat stress induces stress granule formation in both perinuclear regions and the rachis of the *C. elegans* germline, suggesting that stress responses involve coordinated remodeling of multiple RNA granule compartments [20]. These observations suggest that interactions between stress granules and P-bodies may represent an important aspect of cellular stress adaptation.

This study aims to investigate the role of GTBP-1 in regulating reproductive fitness under different temperature conditions and to elucidate how heat stress-induced GTBP-1 enrichment and granule dynamics are regulated in the *C. elegans* germline. Through a combination of genetic approaches, cell biological analyses, and RNAi-based screening, we characterize the domain-specific functions of GTBP-1, its interactions with stress granule and P-body components, and the factors that regulate heat stress-induced GTBP-1 recruitment and enrichment in germline granules. We further assess the regulatory influence of RSKS-1 on GTBP-1 granule dynamics and reproductive homeostasis. Together, our findings provide new insights into how temperature stress influences germline granule dynamics and reproductive fitness in *C. elegans*.

## Results

### Heat stress induces GTBP-1–positive granules in the *C. elegans* germline

*C. elegans* GTBP-1 is the ortholog of mammalian G3BP1, a well-established core component of stress granules. In mammalian systems, G3BP1 is a key determinant of stress granule assembly and contains multiple conserved domains, including a Nuclear Transport Factor 2 (NTF2) domain, an intrinsically disordered region (IDR), an RNA recognition motif (RRM), and an arginine/glycine-rich (RGG) domain [11,15,30]. These domains have been described as characteristic structural features of G3BP1 family proteins. G3BP1 is widely used as a marker of stress granules and is required for their efficient formation under various stress conditions. Based on this conservation, GTBP-1 is commonly used as a stress granule marker in *C. elegans* and has been employed to monitor stress-induced granule dynamics in the germline [31].

To elucidate the functions of GTBP-1 in the germline, we first generated full-length *gtbp-1* knockout alleles (*ust600* and *ust601*) using CRISPR/Cas9 technology (Fig 1A). The *gtbp-1(ust600)* deleted most of the amino acid sequences and *gtbp-1(ust601)* deleted nearly the whole gene sequence, which is likely a null allele. Both mutants exhibited no overt developmental defects, but they showed pronounced reproductive abnormalities under different temperature conditions. At 15 ℃ and 20 ℃, *gtbp-1* mutants produced significantly more progeny than N2 animals (Fig 1B), whereas at 25 ℃ they exhibited a severe fertility defect, suggesting that GTBP-1 plays an important role in maintaining reproductive homeostasis across different temperature conditions. To further characterize the reduced fertility phenotype of *gtbp-1* mutants at 25 ℃, we visualized germline and embryonic development using GFP::HIS-58. Although the germline appeared largely intact, embryos were rarely observed in adult *gtbp-1* mutants at 25 ℃ (S1A Fig), suggesting that the severe fertility defect is associated with impaired embryo production rather than gross loss of germline tissue.

**Fig 1:**
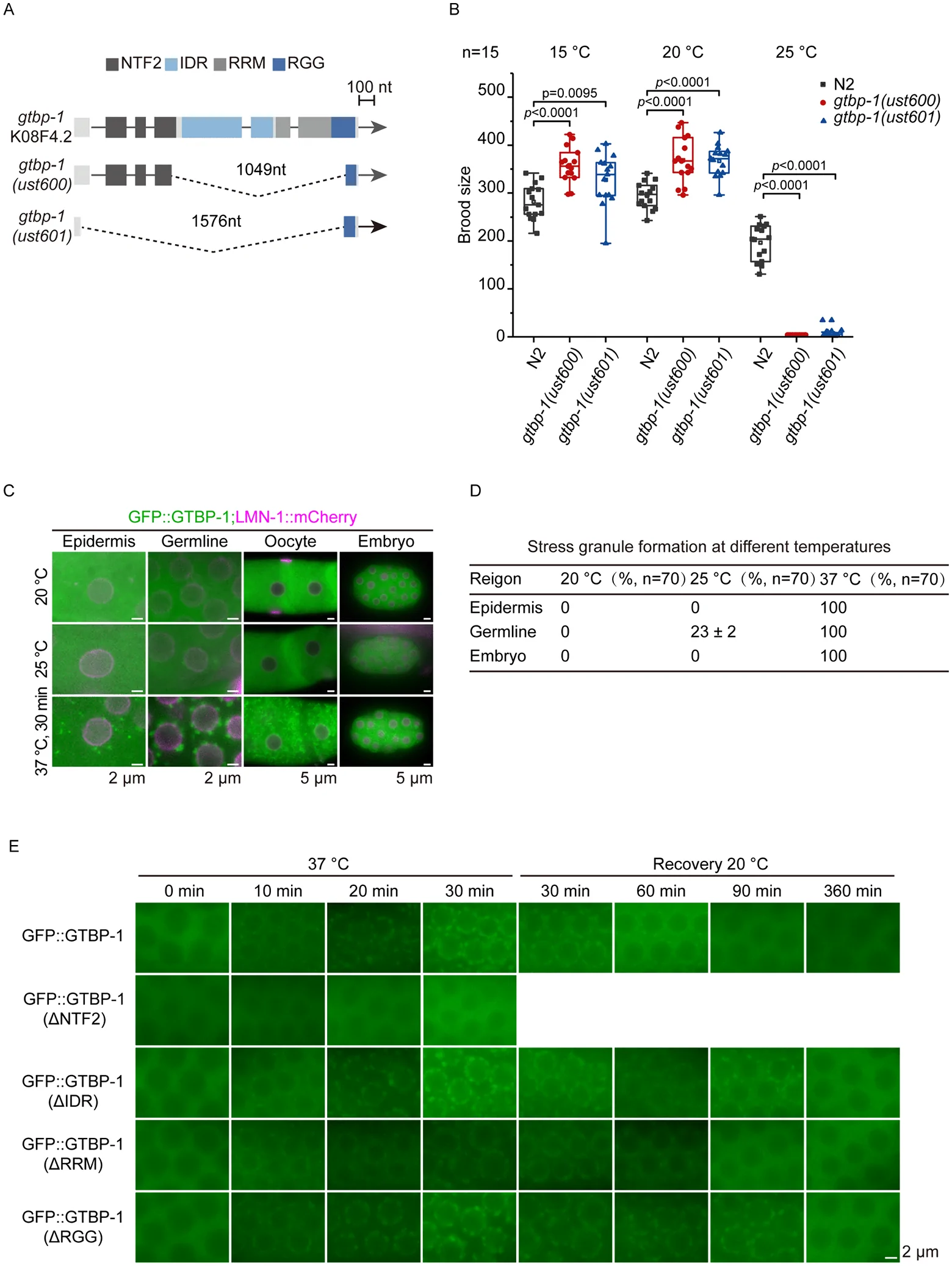
GTBP-1 is required for reproductive homeostasis and temperature-dependent stress granule formation. (A) Schematic representation of the *gtbp-1* gene structure and CRISPR/Cas9-generated deletion alleles (*ust600, ust601*). Domains: Nuclear Transport Factor 2 (NTF2) domain, internal disorder region (IDR) domain, RRM and Arginine/Glycine-rich (RGG) domains. (B) Brood size analysis of *gtbp-1* mutants at 15 ℃, 20 ℃, and 25 ℃, respectively (n = 15 animals per genotype per condition). Box plots show median, 25–75% percentiles, and minimum/maximum values. Statistical significance was determined using two-tailed unpaired Student’s t-test. (C) Representative images of GFP::GTBP-1 localization in multiple tissues under different temperature conditions. GTBP-1 displays diffuse cytoplasmic distribution at 20 ℃ but relocalizes into perinuclear granules upon heat stress (25 ℃ chronic exposure or 37 ℃ acute heat shock for 30 min). LMN-1::mCherry was used to label the nuclear envelope. Images are representative of at least three independent experiments, with ≥ 3 animals per condition. (D) Quantification of stress granule formation across tissues and temperatures. Stress granule assembly is enhanced at 25 ℃ in a tissue-specific manner and is strongly induced by acute heat shock (n = 70 animals per genotype per condition). Data are presented as mean ± SEM from three independent experiments.(E) Domain analysis of GTBP-1. Deletion of the NTF2 domain abolishes stress granule assembly, whereas IDR or RGG deletion permits assembly but delays disassembly during recovery. Images are representative of at least three independent experiments, with ≥ 3 animals per condition.

We generated GFP-tagged GTBP-1 and examined GFP::GTBP-1 distribution across multiple tissues under different temperature conditions (Figs 1C–1D and S1B–S1E). The nuclear envelope was labeled by the lamin protein LMN-1::mCherry. Under normal laboratory conditions (20 ℃), GFP::GTBP-1 exhibited a largely diffuse cytoplasmic distribution in epidermal cells, germ cells, and early embryos, with no obvious granule formation. Analysis of full-germline images further showed that GTBP-1 was broadly distributed throughout both perinuclear regions and the rachis (S1B and S1E Fig).

When animals were cultured at 25 ℃, GTBP-1–positive puncta were detected in the germline of a subset of animals, whereas no GTBP-1–positive puncta were observed in the epidermal cells (Figs 1C and S1D), indicating that prolonged exposure to elevated temperature promotes the formation of GTBP-1–positive puncta in the germline. Upon acute heat shock at 37 ℃ for 30 min, GTBP-1 formed more prominent punctate structures across multiple tissues (Figs 1C and S1B–S1E). In the germline, GTBP-1 enrichment was observed in both perinuclear granules and puncta within the rachis, consistent with previous reports displaying stress granule assembly in this region [20], and was additionally detected in the oocyte cytoplasm (S1B–S1E Fig). These observations reveal that heat stress promotes the redistribution of GTBP-1 into punctate structures throughout the germline, including perinuclear regions and the rachis.

To investigate the domain-specific contributions of GTBP-1 to heat stress-induced granule enrichment and dynamics, we generated transgenic strains expressing GFP::GTBP-1 variants lacking individual protein domains and examined their localization in the germline upon heat shock (Figs 1E and S2A–S2B). Deletion of the NTF2 domain markedly reduced GTBP-1 enrichment in heat stress-induced granules, consistent with previous studies demonstrating an important role of the NTF2-like domain in mammalian G3BP proteins [14,32]. In contrast, deletion of the IDR, RRM, or RGG domain did not substantially alter GTBP-1 enrichment during heat stress, whereas granule clearance during recovery from heat shock was markedly delayed in the ΔIDR and ΔRGG mutants, but not the ΔRRM mutant, suggesting that these domains make distinct contributions to granule dynamics. Analysis of full-germline images showed that the effects of domain deletions in the rachis were consistent with those observed in perinuclear regions (S2B Fig). In addition, all four domain-deletion mutants exhibited reduced fertility at 25 ℃, with deletion of the IDR, RRM, or RGG domain causing particularly severe fertility defects at 25 ℃ (S2C–S2D Fig).

Together, these results show that heat stress induces GTBP-1–positive granules throughout the germline, including both perinuclear regions and the rachis. Different GTBP-1 domains make distinct contributions to granule recruitment and dynamics. Notably, GTBP-1 granule phenotypes did not fully account for the reproductive fitness phenotypes observed at 25 ℃, suggesting that GTBP-1–dependent reproductive function cannot be fully explained by GTBP-1 enrichment or granule dynamics alone.

### GTBP-1 differentially colocalizes with PQN-59 and TIAR-1 in heat stress-induced germline granules

PQN-59 and TIAR-1 are two well-characterized stress granule proteins implicated in stress granule assembly in *C. elegans* [13,18]. PQN-59 is an ortholog of human UBAP2 (ubiquitin associated protein 2) and UBAP2L (ubiquitin associated protein 2 like) [18,33]. TIAR-1 is a member of the TIA-1/TIAR family of RNA-binding proteins and has been reported to localize to germ granules and accumulate in heat stress-induced granules in the *C. elegans* germline [20,34]. We examined the spatial relationship between GTBP-1 and these stress granule proteins following heat shock. At the normal laboratory cultivation temperature at 20 ℃, PQN-59 is evenly distributed in the cytoplasm in epidermis, germline cells, and early and late embryos. Upon heat shock at 37 ℃ for 30 min, PQN-59 accumulated in heat stress-induced perinuclear granules and extensively overlapped with GFP::GTBP-1 signals (Fig 2A–2B). Quantitative colocalization analysis yielded a Pearson’s correlation coefficient of approximately 0.9, confirming strong spatial correlation between GTBP-1 and PQN-59 within germline granules (Fig 2E). In epidermis and early and late embryos, GTBP-1 and PQN-59 granules colocalized in the cytoplasm (S3A Fig).

**Fig 2:**
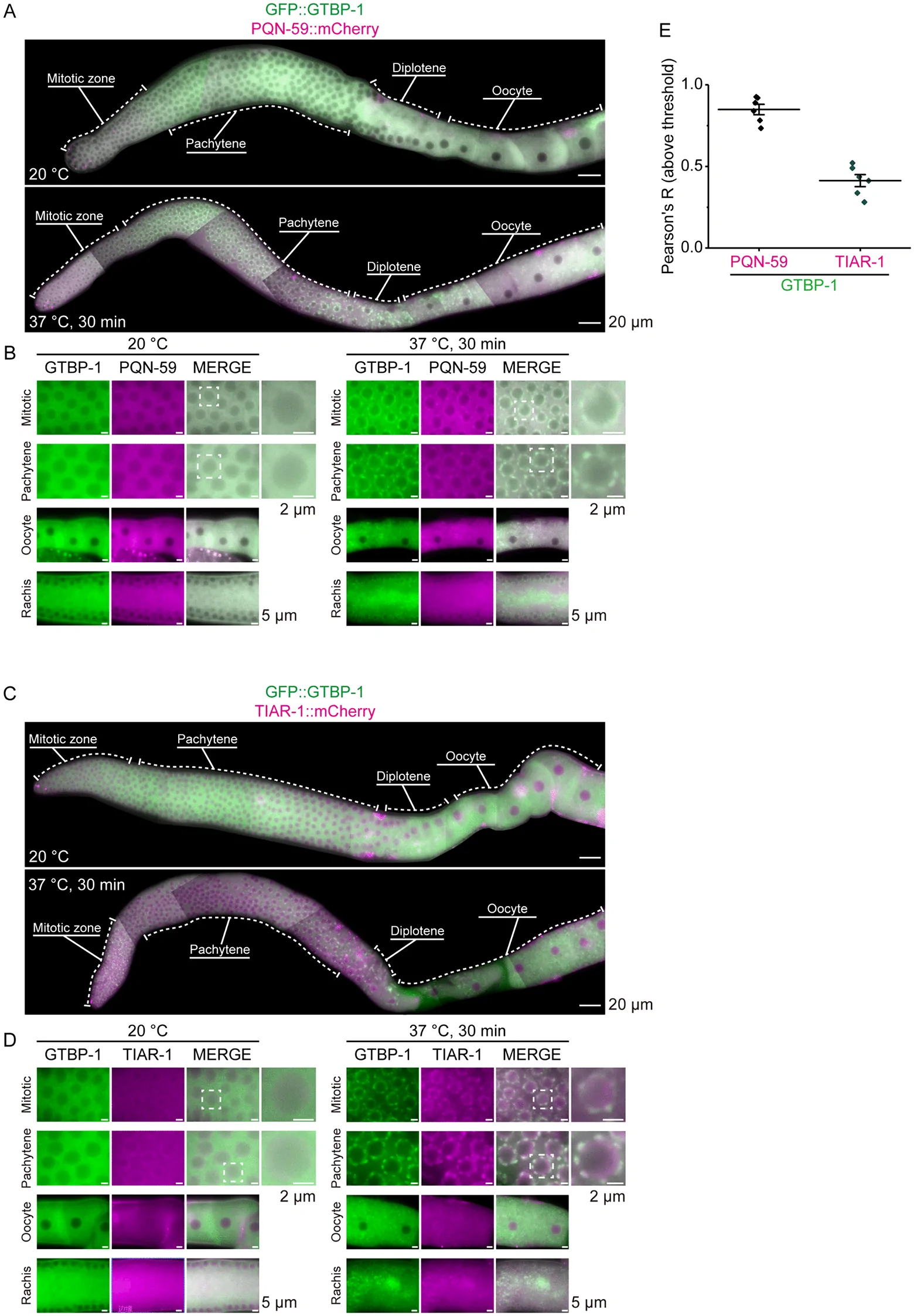
GTBP-1 shows colocalization with PQN-59 and TIAR-1. (A) Representative confocal images of germlines co-expressing GFP::GTBP-1 (green) and the P-body marker PQN-59::mCherry (magenta) at 20 ℃ or after acute heat shock (37 ℃ for 30 min). Germline regions including the mitotic zone, pachytene, diplotene, and oocyte are indicated. Images are representative of at least three independent experiments, with ≥ 3 animals per condition. (B) Magnified views of GFP::GTBP-1 and PQN-59::mCherry localization in the mitotic zone, pachytene stage, oocytes, and rachis under control (20 ℃) and acute heat shock (37 ℃) conditions. Images are representative of at least three independent experiments, with ≥ 3 animals per condition. (C) Representative confocal images of germlines co-expressing GFP::GTBP-1 (green) and the stress granule marker TIAR-1::mCherry (magenta) under control (20 ℃) and acute heat shock (37 ℃ for 30 min). Germline regions including the mitotic zone, pachytene, diplotene, and oocyte are indicated. Images are representative of at least three independent experiments, with ≥ 3 animals per condition. (D) Magnified views of GFP::GTBP-1 and TIAR-1::mCherry localization in the mitotic zone, pachytene stage, oocytes, and rachis under control (20 ℃) and acute heat shock (37 ℃) conditions. Images are representative of at least three independent experiments, with ≥ 3 animals per condition. (E) Quantification of Pearson’s correlation coefficients between GFP::GTBP-1 and PQN-59::mCherry or TIAR-1::mCherry under heat shock conditions. Data are presented as mean ± SEM from at least three independent experiments (n=6 animals per condition).

In contrast, at 20 ℃, TIAR-1 was distributed in both the cytoplasm and nucleoplasm. Upon 37 ℃ heat shock, TIAR-1 was enriched in the nucleus and also remained detectable in cytoplasmic punctate structures in embryos and epidermal cells (S3B Fig). In the germline, both TIAR-1 and GTBP-1 were detected in heat stress-induced granules following heat shock (Fig 2C–2D). Within perinuclear granule-containing regions, GTBP-1 and TIAR-1 showed spatial overlap. Quantitative colocalization analysis performed specifically within these granule-containing regions yielded a Pearson’s correlation coefficient of approximately 0.4 (Fig 2E), indicating a spatial correlation between GTBP-1 and TIAR-1.

### RNAi-based candidate screening identifies factors that regulate heat stress-induced GTBP-1 granules in the germline

Previous studies reported that PQN-59 is dispensable for stress granule assembly in *C. elegans* embryos and that disruption of PQN-59 promotes GTBP-1 enrichment in germ granules under non-stress conditions [18]. To determine whether PQN-59 and TIAR-1 contribute to heat stress-induced GTBP-1 enrichment in the germline, we depleted these factors by RNAi and examined GTBP-1 localization following heat shock.

Although both PQN-59 and TIAR-1 exhibited spatial association with GTBP-1 following heat stress, their functional contributions were distinct. RNAi depletion of *pqn-59* significantly reduced both GTBP-1 granule enrichment and granule number in the germline following heat shock, whereas *tiar-1* RNAi produced little detectable effect (Fig 3A–3C). These results suggest that PQN-59 contributes to heat stress-induced GTBP-1 enrichment in the germline, whereas TIAR-1 appears largely dispensable under these conditions. Consistent with previous observations [18], depletion of *pqn-59* under normal laboratory culture conditions (20 ℃) promoted GTBP-1 enrichment in perinuclear granules of the embryonic germ cells (Fig 3A).

**Fig 3:**
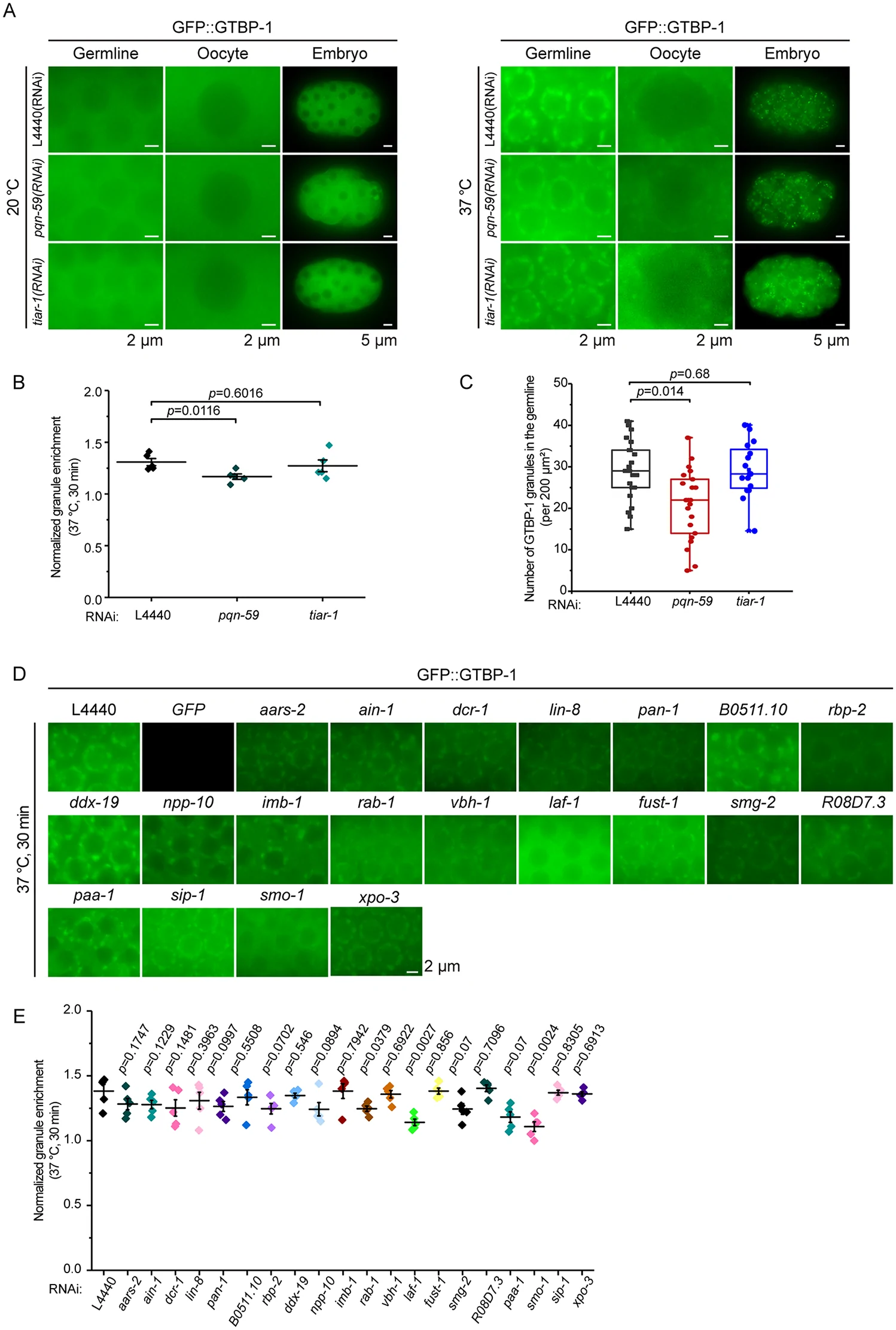
RNAi screening identifies PQN-59, LAF-1, and SMO-1 as regulators of GTBP-1–associated stress granule formation. (A) Fluorescence images of animals expressing GFP::GTBP-1 (green) in the germline, oocytes, and embryos under RNAi conditions at 20 ℃ (left panels) or acute heat shock at 37 ℃ for 30 min (right panels). Images are representative of at least three independent experiments, with ≥ 3 animals per condition. (B) Quantification of GTBP-1 granule enrichment in the germline of animals treated with L4440, *pqn-59*, or *tiar-1* RNAi after acute heat shock (37 ℃ for 30 min). Granule enrichment was normalized to the surrounding cytoplasmic signal. Data are presented as mean ± SEM (n = 5 animals per condition). Each data point represents one animal. Statistical significance was determined using a two-tailed unpaired Student’s t-test. Representative results from at least three independent experiments are shown. (C) Number of GTBP-1 granules per 200 μm² in the germline under the same RNAi conditions as in (B). Box plots show median, 25–75% percentiles, and minimum/maximum values (sampled from ≥ 10 animals per condition). Statistical significance was determined using two-tailed unpaired Student’s t-test. (D) RNAi screen of multiple candidate genes showing their effects on GFP::GTBP-1 granules in the germline under acute heat shock (37 ℃ for 30 min). Images are representative of at least three independent experiments, with ≥ 3 animals per condition. (E) Quantification of GTBP-1 granule enrichment in the germline of animals treated with (D). Granule enrichment was normalized to the surrounding cytoplasmic signal. Data are presented as mean ± SEM (n = 5 animals per condition). Each data point represents one animal. Statistical significance was determined using a two-tailed unpaired Student’s t-test. Representative results from at least three independent experiments are shown.

To identify additional factors involved in heat stress-induced GTBP-1 granule accumulation in the germline, we performed an RNAi-based candidate screen targeting known stress granule components and predicted GTBP-1–interacting proteins [14,32,35,36]. A total of 35 candidate genes were individually depleted by RNAi. Among the genes examined, depletion of *laf-1* and *smo-1* produced the strongest effects on GTBP-1 granules, markedly reducing GTBP-1 enrichment in perinuclear granules following heat shock (Fig 3D–3E). LAF-1 is a DDX3-family RNA helicase that localizes to both the cytoplasm and germline granules and has been implicated in asymmetric phase separation and germ cell fate determination [24,37,38]. SMO-1 is the *C. elegans* ortholog of SUMO1 (small ubiquitin-like modifier 1), suggesting that SUMOylation may contribute to the regulation of GTBP-1 granules during heat stress. Western blot analysis showed that depletion of *pqn-59*, *tiar-1*, *laf-1*, or *smo-1* did not substantially alter total GTBP-1 protein levels (S6 Fig), indicating that the observed granule phenotypes are not attributable to changes in total GTBP-1 abundance.

Because LAF-1 and SMO-1 strongly influenced heat stress-induced GTBP-1 granules, we next examined whether depletion of these factors affected the fertility phenotype of *gtbp-1* mutants at 25 ℃. No rescue of the fertility defect was observed following *laf-1* or *smo-1* RNAi treatment (S4 Fig), indicating that depletion of these factors is insufficient to restore the fertility of *gtbp-1* animals. Together, these results identify PQN-59, LAF-1, and SMO-1 as important regulators of heat stress-induced GTBP-1 granules in the germline.

### GTBP-1 preferentially associates with P-bodies among germ granule compartments

Our recent work identified seven distinct perinuclear germ granule compartments in the *C. elegans* germline. To assess the spatial relationship between heat shock-induced GTBP-1 granule and these germ granules, we crossed GFP::GTBP-1 with the seven tagRFP or mCherry-tagged germ granule strains (Fig 4A–4B). Among the marker proteins, PGL-1 stands for P granules, CGH-1 for P-bodies, ZNFX-1 for Z granules, MUT-16 for Mutator foci, SIMR-1 for SIMR foci, ELLI-1 for E granules and DDX-19 for D granules [25,26,39–42].

**Fig 4:**
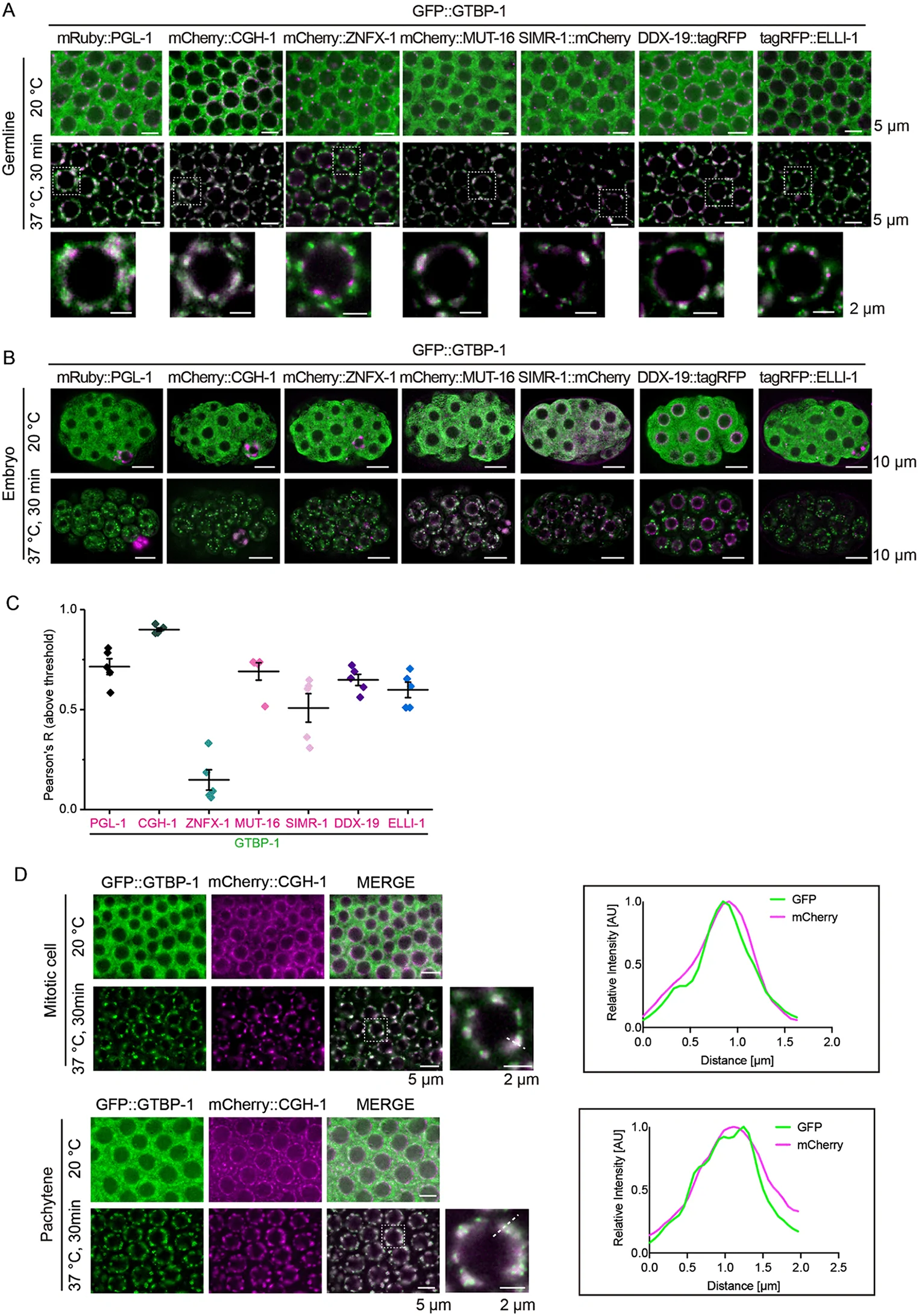
GTBP-1 preferentially associates with P-bodies among germ granule compartments. (A–B) Subcellular colocalization assay of GFP::GTBP-1 with seven germ granule markers (mRuby::PGL-1 for P granule, mCherry::CGH-1 for P-body, mCherry::ZNFX-1 for Z granule, mCherry::MUT-16 for mutator foci, SIMR-1::mCherry for SIMR granule, DDX-19::tagRFP for D granule, and tagRFP::ELLI-1 for E granule) in the germline and early embryos at 20 ℃ or following heat shock (37 ℃ for 30 min). Images are representative of at least three independent experiments, with ≥ 3 animals per condition. (C) Quantification of Pearson’s correlation coefficients between GFP::GTBP-1 and seven germ granule markers under heat stress conditions. Data are presented as mean ± SEM from at least three independent experiments (n = 5 animals per condition). (D) Colocalization of GFP::GTBP-1 (green) with mCherry::CGH-1 (magenta) in mitotic cells of the germline (top panels) and meiotic cells of the germline (bottom panels). Fluorescence intensity along the dotted line was quantified using ImageJ. Images are representative of at least three independent experiments, with ≥ 3 animals per condition.

Following heat shock (37 ℃ for 30 min), GTBP-1 was detected in close association with multiple perinuclear germ granule compartments (Fig 4A). To quantitatively evaluate these relationships, we measured Pearson’s correlation coefficients between GTBP-1 and each germ granule marker across entire images (Fig 4C). Among the seven germ granule markers examined, GTBP-1 exhibited the highest Pearson’s correlation with the P-body marker CGH-1 under heat stress. GTBP-1 also showed moderate-to-high correlations with PGL-1, MUT-16, DDX-19, and ELLI-1, whereas the correlation with ZNFX-1 was substantially lower. Similar associations between GTBP-1 and CGH-1 were observed in both the mitotic and meiotic regions of the germline, as supported by line-scan analyses across representative granules (Figs 4D and S5A). A similar relationship was observed in early embryos, where heat stress-induced GTBP-1 granules were closely associated with CGH-1-positive granules throughout the two-cell, four-cell, and early multicellular stages (S5B Fig).

To examine whether P-body components influence heat stress-induced GTBP-1 enrichment, we depleted several core P-body components, including *cgh-1*, *edc-3* and *ifet-1*, which encode conserved factors involved in P-body assembly and mRNA metabolism, by feeding RNAi [43,44]. Consistent with their roles in P-body formation, depletion of *edc-3* and *ifet-1* disrupted the perinuclear CGH-1–positive P-body structures in the germline. We then quantified GTBP-1 granule enrichment following depletion of these P-body components. RNAi-mediated depletion of *cgh-1*, *edc-3*, or *ifet-1* significantly reduced normalized GTBP-1 enrichment after heat shock (Fig 5A–5B).

**Fig 5:**
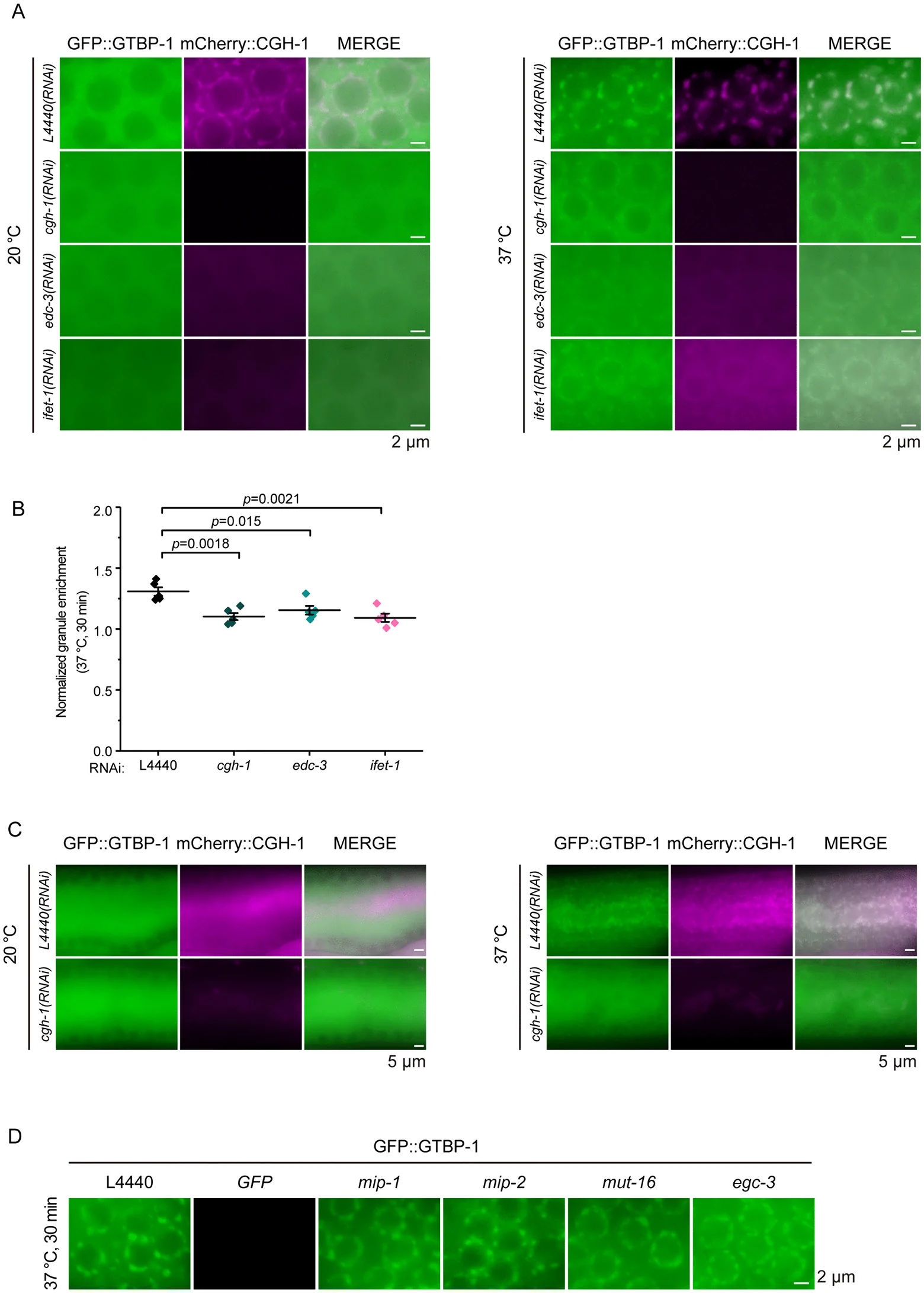
Germline P-body components are required for efficient heat stress-induced GTBP-1 granule assembly. (A) Representative fluorescence images showing GFP::GTBP-1 and mCherry::CGH-1 subcellular localization in gravid adult germlines under RNAi against *cgh-1*, *edc-3*, or *ifet-1*. Images are representative of at least three independent experiments, with ≥ 3 animals per condition. (B) Quantification of GTBP-1 granule enrichment in the germline of animals treated with L4440, *cgh-1*, *edc-3*, or *ifet-1* RNAi after acute heat shock (37 ℃ for 30 min). Granule enrichment was normalized to the surrounding cytoplasmic signal. Data are presented as mean ± SEM (n = 5 animals per condition). Each data point represents one animal. Statistical significance was determined using a two-tailed unpaired Student’s t-test. Representative results from at least three independent experiments are shown. (C) Representative confocal images of the germline rachis co-expressing GFP::GTBP-1 (green) and mCherry::CGH-1 (magenta) in animals treated with L4440 or *cgh-1* RNAi under control conditions (20 ℃) or acute heat shock (37 ℃ for 30 min). Images are representative of at least three independent experiments, with ≥ 3 animals per condition. (D) RNAi screen of multiple candidate genes showing their effects on GFP::GTBP-1 granules in the germline under acute heat shock (37 ℃ for 30 min). Images are representative of at least three independent experiments, with ≥ 3 animals per condition.

To address the possibility that reduced perinuclear GTBP-1 enrichment might reflect redistribution of GTBP-1 to other germline regions, we further examined GTBP-1 localization in full-germline following *cgh-1* RNAi. In addition to disrupting perinuclear GTBP-1 granules, *cgh-1* depletion also markedly impaired heat stress-induced GTBP-1 enrichment in granules within the rachis (Fig 5C), further supporting a contribution of P-body organization to heat stress-induced GTBP-1 enrichment across germline compartments.

Western blot analysis confirmed that depletion of *cgh-1*, *edc-3*, or *ifet-1* did not substantially alter total GTBP-1 protein levels (S6 Fig), indicating that the observed granule phenotypes were not caused by reduced GTBP-1 abundance.

To determine whether GTBP-1 granule formation depends on other germ granule components, we examined GTBP-1 localization following depletion of *mip-1*, *mip-2*, *mut-16*, or *egc-3*. MIP-1 and MIP-2 are required for the perinuclear anchoring of P and Z granules, MUT-16 and EGC-3 are required for the assembly of mutator foci and the E granule respectively [24,45]. Upon knocking down *mip-1*, *mip-2*, *mut-16*, and *egc-3* by feeding RNAi, GTBP-1 was still recruited to perinuclear condensates in germline (Fig 5D), suggesting that GTBP-1 puncta formation does not depend on P, Z, M, and E-germ granule components.

Together, these findings suggest that GTBP-1 exhibits the strongest association with perinuclear P-bodies following heat stress and that P-body organization contributes to heat stress-induced GTBP-1 condensate enrichment.

### Forward genetic screening identifies RSKS-1 as a regulator of temperature-dependent fertility and heat stress-induced GTBP-1 granule dynamics

To investigate the relationship between heat stress-induced GTBP-1 granules and reproductive fitness, we performed a forward genetic screen to search for mutants that rescue the fertility defect of *gtbp-1* mutants at 25 ℃. From approximately 20,000 haploid genomes, we isolated a suppressor mutant, *rsks-1(ust761)*, that restored fertility in *gtbp-1(ust600)* animals (Fig 6A–6B). The *rsks-1(ust761)* allele carries a histidine-to-asparagine substitution at amino acid 200. To validate this finding, we further analyzed another *rsks-1* deletion allele *(ok1255)*, generated an additional CRISPR-Cas9 allele *(ust763)*, and performed *rsks-1* RNAi knockdown. Importantly, loss of *rsks-1* consistently suppressed the fertility defect of *gtbp-1* mutants at 25 ℃, restoring brood size to a level comparable to that of the *rsks-1(ok1255)* single mutant (Fig 6B).

**Fig 6:**
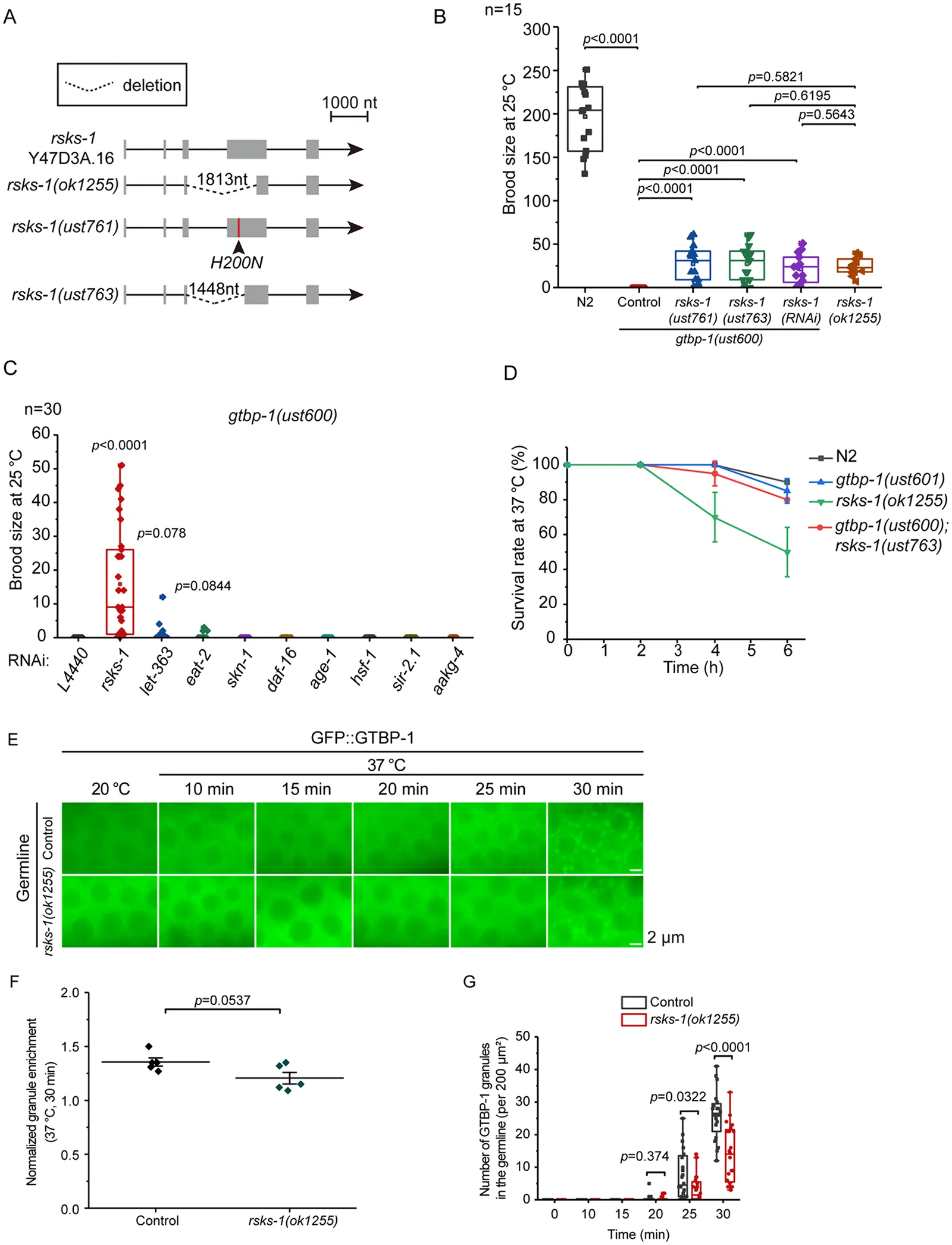
RSKS-1 promotes GTBP-1 granule recruitment during heat stress and supports germline reproductive homeostasis. (A) Schematic representation of the *rsks-1* gene structure and the alleles (*ok1255, ust761, ust763*) generated by CRISPR-Cas9 and chemical mutagenesis. (B) Brood size analysis of N2, *gtbp-1* mutant, *rsks-1* mutant, and *rsks-1;gtbp-1* double mutants at 25 ℃ (n=15 animals per genotype). Box plots show median, 25–75% percentiles, and minimum/maximum values. Statistical significance was determined using two-tailed unpaired Student’s t-test. (C) Brood size analysis of *gtbp-1* mutants at 25 ℃ following RNAi knockdown of selected lifespan-regulating genes (n=30 animals per genotype). Box plots show median, 25–75% percentiles, and minimum/maximum values. Statistical significance was determined using two-tailed unpaired Student’s t-test. Groups with complete infertility were not subjected to statistical analysis. (D) Survival curves of various experimental groups at 37 ℃. Data represent mean ± SEM from three independent experiments; error bars indicate SEM. (E) Expression of GFP::GTBP-1 in the germline of wild-type (control) and *rsks-1(ok1255)* worms following 37 ℃ heat shock. Images are representative of at least three independent experiments, with ≥ 3 animals per condition. (F) Quantification of normalized GTBP-1 granule enrichment in the germline of control and *rsks-1(ok1255)* animals following acute heat shock (37 ℃ for 30 min), corresponding to the images shown in (E). Granule enrichment was normalized to the surrounding cytoplasmic signal. Data are presented as mean ± SEM (n = 5 animals per condition). Each data point represents one animal. Statistical significance was determined using a two-tailed unpaired Student’s t-test. Representative results from at least three independent experiments are shown. (G) Number of GTBP-1 granules per 200 μm² in the germline under the same conditions as in (E). Box plots show median, 25–75% percentiles, and minimum/maximum values (sampled from ≥10 animals). Statistical significance was determined using two-tailed unpaired Student’s t-test. Statistical analysis was not performed for groups in which no detectable granules were observed.

In mammalian cells, S6K localizes to oxidative stress–induced stress granules and contributes to their assembly and maintenance [46]. RSKS-1, the *C. elegans* homolog of S6K and a key effector of the mTOR pathway, is known to regulate translation and lifespan [47]. To determine whether suppression of *gtbp-1* sterility reflected a general reduction in mTOR signaling or longevity pathways, we depleted multiple well-characterized lifespan-regulatory genes by RNAi [48–50]. None of these treatments rescued the fertility defect of *gtbp-1* mutants at 25 ℃ (Fig 6C). From the forward genetic screen, we only recovered a *rsks-1* allele that reproducibly suppressed the fertility defects of *gtbp-1* mutant.

Heat-shock survival assays revealed a similar reduction of survival between *gtbp-1* and wild type N2 animals. Interestingly, the mutation of *rsks-1* further promoted the heat shock-induced lethality than wild type N2 animals (Fig 6D). The *rsks-1; gtbp-1* double mutant revealed similar survival to that of wild type N2 animals upon heat shock, suggesting that RSKS-1 and GTBP-1 may counteract each other in mediating stress reactions.

Because RSKS-1 influenced GTBP-1–dependent fertility, we next examined whether it also affects heat stress-induced GTBP-1 granules in the germline. Time-course analysis revealed that loss of *rsks-1* delayed the appearance of GTBP-1 granules and reduced granule number following heat shock and significantly reduced granule number throughout the heat stress period (Fig 6E and 6G). In contrast, the average GTBP-1 enrichment of heat stress-induced granules was not significantly altered (Fig 6F). These results suggest that RSKS-1 primarily affects the kinetics and abundance of heat stress-induced GTBP-1 granules rather than the extent of GTBP-1 enrichment within individual granules. Western blot analysis showed that *rsks-1* RNAi did not substantially affect total GTBP-1 protein levels (S6 Fig), indicating that the observed granule defects are unlikely to result from reduced GTBP-1 expression.

To further investigate how RSKS-1 participates in heat stress responses, we examined its subcellular localization before and after heat shock. LMN::mCherry was used to display the nuclear membrane. Under normal laboratory conditions (20 ℃), RSKS-1 was broadly distributed throughout the cytoplasm and nucleoplasm. Following heat shock (37 ℃ for 30 min), RSKS-1 underwent a striking relocalization and accumulated in discrete perinuclear granule-like structures in germline and oocyte. In embryos, RSKS-1 was enriched in the nucleus and formed puncta in the cytoplasm (Fig 7A). Because GTBP-1 exhibited its strongest association with P-bodies following heat stress, we next examined the relationship between RSKS-1 and the P-body marker CGH-1. Heat-induced RSKS-1 granules showed clear spatial overlap with CGH-1-positive structures in the germline (Fig 7B–7C). Quantitative colocalization analysis within perinuclear granule-containing regions yielded a mean Pearson’s correlation coefficient of 0.674, indicating a positive spatial correlation between RSKS-1 and CGH-1 (Fig 7D).

**Fig 7:**
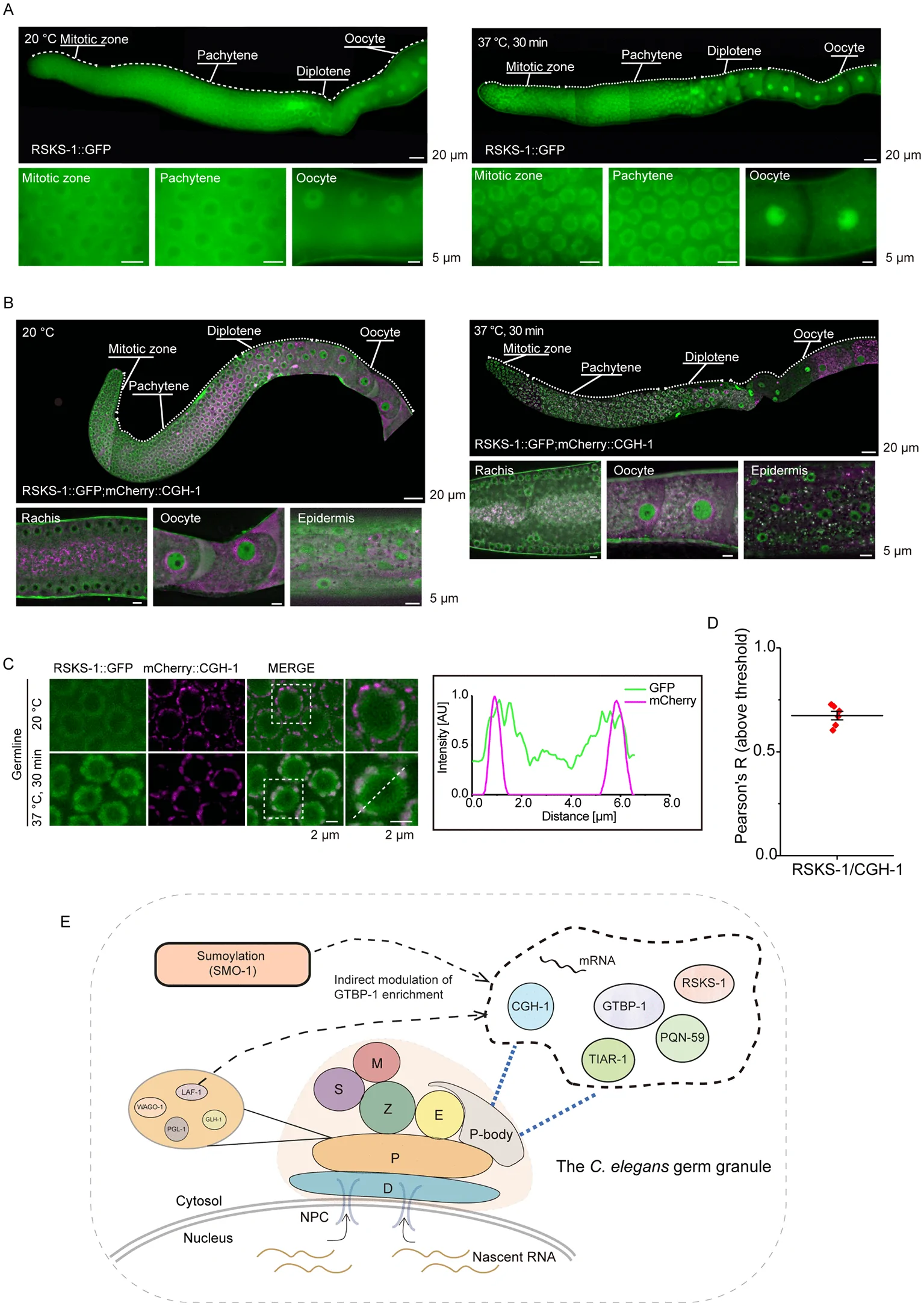
Heat stress induces perinuclear enrichment of RSKS-1 in P-body. (A) Representative images of RSKS-1::GFP localization in multiple tissues at 20 ℃ and following heat shock (37 ℃ for 30 min). Images are representative of at least three independent experiments, with ≥ 3 animals per condition. (B) Representative confocal images of germlines co-expressing RSKS-1::GFP (green) and the P-body marker mCherry::CGH-1 (magenta) under control conditions (20 ℃, left panels) or after acute heat shock (37 ℃ for 30 min, right panels). Major germline regions, including the mitotic zone, pachytene, diplotene, and oocytes, are indicated. Higher-magnification views of the rachis, oocytes, and epidermis are shown below. Images are representative of at least three independent experiments, with ≥ 3 animals per condition. (C) Representative images of the subcellular localization of RSKS-1::GFP (green) and the P-body marker mCherry::CGH-1 (magenta) in the germline under culture conditions (20 ℃) or acute heat shock (37 ℃ for 30 min). Fluorescence intensity along the dotted line was quantified using ImageJ. Images are representative of at least three independent experiments, with ≥ 3 animals per condition. (D) Quantification of Pearson’s correlation coefficients between RSKS-1::GFP and mCherry::CGH-1 within perinuclear granules under heat stress conditions. Data are presented as mean ± SEM from at least three independent experiments (n = 6 animals per condition). (E) A working model of heat stress-induced GTBP-1 condensate enrichment in perinuclear P-body-associated granules. The *C. elegans* germline contains approximately seven perinuclear germ granules, among which P-bodies are marked by CGH-1, EDC-3, and IFET-1. Upon heat stress, PQN-59, TIAR-1, RSKS-1, and GTBP-1 enrichment in perinuclear germline granules, with GTBP-1 showing a strong spatial association with P-body markers.

Together, these findings identify RSKS-1 as a heat-responsive regulator of GTBP-1 granule dynamics and GTBP-1–dependent fertility under elevated temperature conditions. The association of RSKS-1 with P-bodies further supports a role for RSKS-1 in the heat stress response of the germline.

## Discussion

Stress granules have been widely recognized as key regulators of cellular stress responses, yet their germline-specific assembly and physiological significance remain incompletely understood. Stress granules and P-bodies are closely related ribonucleoprotein condensates that frequently interact and exchange components under stress conditions across diverse organisms, suggesting conserved functional links between these compartments [51–53].

Here, using *C. elegans*, we identify GTBP-1 as an important regulator of temperature-dependent reproductive homeostasis. Upon heat stress, GTBP-1 exhibits its strongest association with perinuclear P-bodies in germ cells. Depletion of PQN-59, LAF-1, SMO-1, RSKS-1, and P-body components reduced heat stress-induced GTBP-1 condensate enrichment in the germline (Fig 7E). Together, these findings suggest that GTBP-1-containing punctate structures represent stress-responsive assemblies associated with P-bodies and may contribute to germline responses to elevated temperature.

An interesting finding is that *gtbp-1* mutant produced significantly more progeny than wild-type animals at 15 ℃ and 20 ℃, yet exhibited severe fertility defects at 25 ℃. Further analysis revealed that the germline of *gtbp-1* mutants remained largely intact at 25 ℃, whereas embryos were rarely observed in adult animals, suggesting that the severe fertility defect is associated with impaired embryo production rather than a gross defect in embryonic development. Previous studies reported that GTBP-1 regulates lifespan and stress resistance in *C. elegans* [18,19,54,55]. Our findings extend the functions of GTBP-1 by identifying its role in maintaining reproductive fitness under temperature stress. The contrasting fertility phenotypes under different temperature conditions indicate that GTBP-1 contributes to the maintenance of reproductive homeostasis across a broad thermal range. Consistent with this notion, mammalian G3BP proteins have been reported to exert context-dependent effects on cell proliferation and stress adaptation [8,11,56,57]. Together, these findings suggest that the physiological functions of GTBP-1 in reproductive regulation cannot be fully explained by its association with stress-responsive granules and may extend more broadly to maintaining reproductive fitness under changing environmental conditions.

Domain analysis revealed distinct contributions of GTBP-1 protein domains to heat stress-induced granule dynamics. Deletion of the NTF2 domain markedly reduced GTBP-1 enrichment in heat stress-induced granules, consistent with previous studies showing an important role of the NTF2-like domain in mammalian G3BP proteins [14,15]. In contrast, deletion of the IDR or RGG region did not substantially impair initial GTBP-1 enrichment in granules but delays granule disassembly during recovery, highlighting their contribution to stress granule fluidity and reversibility [58,59]. These findings suggest that different GTBP-1 domains make distinct contributions to granule formation and disassembly and that granule formation and turnover can be differentially regulated by individual domains. Interestingly, all four domain-deletion mutants exhibited reduced fertility at elevated temperature, suggesting that the physiological functions of GTBP-1 extend beyond its role in heat stress-induced granules. Notably, the severity of the reproductive defects and the extent of granule enrichment of GTBP-1 deletion mutants suggest that GTBP-1–dependent reproductive fitness cannot be simply explained by GTBP-1 enrichment or granule dissolution dynamics. Alternatively, the roles of the IDR, RRM, and RGG regions of G3BP proteins in RNA interactions suggest that these domains may also contribute to GTBP-1–dependent reproductive fitness through RNA-related mechanisms, a possibility remains to be tested [14,15].

The spatial relationships among granule components may reflect the heterogeneous composition of stress granules and other RNA condensates, which can exhibit distinct molecular substructures [4,60–62]. Heat stress-induced GTBP-1 granules were also observed in epidermal cells. This observation suggests that recruitment of GTBP-1 to perinuclear condensates is not unique to the germline. Whether these somatic granules share similar molecular composition, regulatory mechanisms, or functions with germline GTBP-1 granules remains to be determined.

Our RNAi-based candidate screen identified several regulators of heat stress-induced GTBP-1 granules in the germline. Among these factors, depletion of *laf-1* or *smo-1* strongly reduced GTBP-1 enrichment within heat stress-induced granules, whereas depletion of *pqn-59* reduced granule number and enrichment but did not completely abolish heat stress-induced GTBP-1 granules. The effect of LAF‑1 depletion is consistent with its established role as a DEAD-box RNA helicase that regulates RNA condensate assembly and germ granule organization [37,38,63]. Interestingly, although LAF-1 is primarily associated with P granules [37], its depletion significantly reduced GTBP-1 enrichment in heat stress-induced granules. These findings suggest that LAF-1, as a P granule component, may indirectly contribute to GTBP-1 enrichment in P-body-associated condensates, although the underlying mechanism remains unclear. In *C. elegans*, *smo-1* encodes the sole SUMO protein, and SUMOylation is elevated under stress [64–66]. The sensitivity of GTBP-1 granules to *smo-1* depletion therefore suggests that SUMO-dependent mechanisms may contribute to the regulation of GTBP-1 granule formation during heat stress. However, neither factor rescues the sterility of *gtbp-1* mutants at 25 ℃. While these observations indicate that disruption of GTBP-1 granules alone is insufficient to restore fertility, they do not exclude additional developmental or physiological functions for these factors that may also influence reproductive outcomes.

Our study reveals a close spatial relationship between heat stress-induced GTBP-1 granules and P-bodies in the *C. elegans* germline. Among the seven germ granule compartments examined, GTBP-1 exhibited the strongest association with the P-body marker CGH-1 following heat stress. Moreover, disruption of core P-body components impaired heat stress-induced GTBP-1 condensate enrichment, further supporting an association between P-body organization and GTBP-1 localization. Notably, stress granules and P-bodies have been reported to physically and functionally interact across diverse organisms, with several P-body assembly factors being required for efficient stress granule formation in yeast [4,27,31,52,67–70]. Recent studies have further highlighted the complexity of germline P-body-like condensates in *C. elegans*, which coexist and interact with multiple perinuclear RNA granule compartments, including P, Z, E, D, SIMR and Mutator granules [71]. The preferential association of GTBP-1 with CGH-1-positive structures observed in this study is consistent with this organizational framework. The partial spatial overlap observed with other germ granule compartments may reflect the finite spatial resolution of fluorescence imaging and/or stress-induced changes in granule morphology or boundaries. The molecular mechanisms underlying this interaction, including whether SUMOylation contributes to the process, remain to be determined. Although CGH-1 disruption impaired GTBP-1 granule formation in both perinuclear and rachis compartments, whether other granule components contribute to GTBP-1 recruitment in rachis-associated granules remains unclear.

The mechanistic target of rapamycin (mTOR) signaling pathway plays a central role in multicellular organisms by coordinating protein translation, energy metabolism, and lifespan regulation [72–74]. Its downstream effector, ribosomal S6 kinase (S6K), has been shown in mammals to participate in stress granule assembly under oxidative stress. Notably, loss of the *C. elegans* homolog, RSKS-1, attenuates heat stress-induced germline stress granule formation and markedly decreases worm survival [46]. In this study, we identified *rsks-1* as the only suppressor recovered from a forward genetic screen for modifiers of *gtbp-1* fertility defects. Loss or depletion of *rsks-1* suppressed the fertility defect of *gtbp-1* mutants at 25 ℃ and altered heat stress-induced GTBP-1 enrichment. In addition, RSKS-1 underwent striking relocalization following heat stress and accumulated in CGH-1–positive perinuclear granules. These observations support a close spatial association between RSKS-1 and P-body-associated granules and suggest that RSKS-1 participates in the regulation of stress-responsive germline granules and may functionally link mTOR signaling to stress-induced granule dynamics. However, the extent to which RSKS-1 influences the organization of other germ granule compartments, including P granules and germline P-bodies, was not directly examined in the present study and remains an important question for future investigation. Although the mechanisms underlying these effects remain unclear, the genetic interaction between *rsks-1* and *gtbp-1* highlights a previously unrecognized connection between translational regulation, GTBP-1 function, and reproductive fitness under elevated temperature conditions.

In conclusion, our study identifies GTBP-1 as a key regulator of temperature-dependent reproductive fitness in the *C. elegans* germline and supports a spatial association relationship between heat stress-induced GTBP-1 granules and P-bodies. We further identify LAF-1, SMO-1, PQN-59, and RSKS-1 as regulators of GTBP-1 granule enrichment and dynamics, highlighting the contribution of stress-responsive RNA granule organization to the germline response to elevated temperature conditions. Future studies will be required to define the molecular composition of GTBP-1 granules and to determine how interactions among stress granules, P-bodies, and signaling pathways coordinate germline homeostasis during environmental stress.

## Materials and methods

### Strains

The *C. elegans* strains used in this work are listed in S1 Table. Animals were maintained on Nematode Growth Medium (NGM) plates seeded with *E. coli* OP50. All strains were grown at 20 ℃ unless otherwise specified.

### Construction of deletion mutants and transgenic strains

Deletion mutants and transgenic *C. elegans* strains were generated using the CRISPR/Cas9 system. For transgenic strains, coding sequences for GFP::3xFLAG or mCherry fused to a linker (GGAGGTGGAGGTGGAGCT) were inserted either upstream of the stop codon or downstream of the start codon of the target gene. For deletion mutants, sgRNAs were designed to target the coding sequence of the gene of interest. The sgRNA sequences used are listed in S2 Table. Repair plasmids were constructed using the ClonExpress MultiS One Step Cloning Kit (C113-02, Vazyme), and plasmids for injection were prepared using the AxyPrep Plasmid Miniprep Kit (Axygen). The injection mixture contained 50 ng/μl Cas9 vector (pDD162), 50 ng/μl repair plasmid (for knock-in) or no repair template (for deletions), 5 ng/μl co-injection marker plasmid (pCFJ90), and 30 ng/μl each of 2–3 sgRNA plasmids, and was injected into young adult worms. Mutants and knock-in transgenic lines were confirmed by PCR amplification and DNA sequencing, and transgenic lines were further validated by fluorescence microscopy. Sequence of repair plasmids primers are listed in S3 Table.

### Forward genetic screens

We used forward genetic screens to isolate *gtbp-1* suppressors. Basically, the *gtbp-1(ust600)* mutant animals (P0) were synchronized at the late L4 larval stage, collected in 4 mL M9 buffer, and incubated with 50 mM ethyl methanesulfonate (EMS) for 4 hours at room temperature with constant rotation. Animals were then washed with M9 three times and cultured under standard conditions. After 20 hours, adult animals were bleached. Eggs (F1) were distributed and raised on ~100 9-cm NGM plates, resulting in a total of 30–50 plates for the F1 generation, each containing 50–100 eggs. These F1 animals were cultured until gravid, then subjected to bleaching to synchronize the F2 generation. The resulting F2 embryos were distributed onto new plates and incubated at 25 ℃. Putative suppressor mutants were identified in the F3 generation based on their ability to produce viable and fertile progeny at the restrictive temperature of 25 ℃, a phenotype not observed in the original *gtbp-1(ust600)* mutant. F3 animals were individually cloned, and their fertility was further confirmed in the F4 generation to ensure heritability of the suppressor phenotype. The causative mutation was identified through whole-genome sequencing and subsequently confirmed by the acquirement of multiple alleles.

### Brood size

L4 worms were placed individually onto fresh NGM plates. The numbers of progeny that reached the L2 or L3 stage were scored. At least 15 animals per genotype per condition were analyzed in at least three independent experiments.

### Heat stress assays

For chronic mild heat stress: synchronized animals were continuously cultivated at the restrictive temperature of 25 ℃ from the L1 larval stage. Worms at the young adult stage were photographed for subsequent phenotypic analysis. For acute heat shock: synchronized L4 to young adult-stage worms were individually picked onto fresh NGM plates. The plates were sealed and subjected to a heat shock at 37 ℃ for 30 minutes. For recovery experiments, after heat shock plates were immediately returned to 20 ℃ for a specified period before phenotypic assessment or imaging.

### Microscopy and image analysis

For imaging of live adult animals, worms were immobilized in ddH_2_O containing 0.25 M sodium azide and mounted on freshly prepared 2% agarose pads. For high-resolution imaging of embryos and germ cells within adult animals, worms were dissected in 2 µL of 0.4 × M9 buffer containing 0.1 M sodium azide, and the released gonads or embryos were transferred onto freshly prepared 1.1% agarose pads. The Leica TNUNDER Imaging System was used, equipped with a K5 sCMOS microscope camera and an HC PL APO 100x/1.40-0.70 oil objective. Images were taken and deconvoluted using Leica Application Suite X software (version 3.7.4.23463). For all comparative analyses within the same experiment, identical imaging parameters were maintained, including laser power, exposure time, gain, and deconvolution settings. Image brightness and contrast were adjusted uniformly across all samples within each experiment.

### Stress granule quantification

Stress granules were quantified in the meiotic region of adult germlines. Regions of interest (ROIs; ~ 200 μm^2^) were defined within comparable anatomical regions. For each condition, at least 10 independent animals were analyzed, with 1–2 ROIs per animal. ROI selection was performed in a blinded manner.

Stress granules were defined as discrete cytoplasmic foci exhibiting fluorescence intensity above a consistent threshold applied uniformly across all samples within each experiment. Granule number was quantified by counting all threshold-positive granules within each predefined ROI and expressed as the number of granules per 200 μm^2^. For granule enrichment analysis, 10 stress granules were randomly selected for intensity measurement. A total of five independent animals were analyzed per condition. Granule enrichment was quantified using ImageJ by measuring the mean fluorescence intensity within segmented granules and adjacent cytoplasmic regions. Background fluorescence was subtracted from both granule and cytoplasmic intensities, and the background-corrected granule intensity was normalized to the background-corrected cytoplasmic intensity.

### Colocalization analysis

Colocalization between fluorescently tagged proteins was quantified using the Coloc2 plugin in ImageJ. For each germline, five ROIs (~200 μm^2^ each) were selected from perinuclear regions. Pearson’s correlation coefficients were calculated for each ROI and averaged within each animal; the resulting animal-level mean was used for statistical analysis. At least five independent animals were analyzed per condition. All analyses were performed using identical threshold settings across samples within each experiment. Background subtraction was applied prior to analysis. For GTBP-1/TIAR-1 and RSKS-1/CGH-1 colocalization analysis, 20 randomly selected perinuclear granules were analyzed per animal. Pearson’s correlation coefficients were calculated for each granule. Line-scan profiles across individual granules were generated using the ImageJ Plot Profile function.

### RNAi screening

RNAi experiments were performed by feeding worms with *E. coli* HT115(DE3) expressing double-stranded RNA targeting specific genes. The RNAi bacterial clones were obtained from the Ahringer feeding library and confirmed by Sanger sequencing. For analysis of stress granule dynamics, synchronized L1 larvae were raised on RNAi plates at 20 ℃ until the L4 or young adult stage, and then subjected to heat shock at 37 ℃ for 30 minutes. For brood size analysis, synchronized L1 larvae were raised on RNAi plates and maintained at the restrictive temperature of 25 ℃ throughout development until progeny counting. In all experiments, worms fed with the empty vector L4440 were used as the RNAi control. A list of the genes used in feeding RNAi experiments is provided in S4 Table.

### Western blot analysis

Synchronized L1 larvae were subjected to RNAi feeding and cultured until the young adult stage. Worms were collected and washed extensively with M9 buffer to remove bacteria prior to protein extraction. For Western blot analysis, protein samples were separated by SDS-PAGE and transferred onto PVDF membranes. Membranes were blocked and incubated with primary antibodies against FLAG to detect tagged proteins. β-actin was used as a loading control. For RNAi-related analyses, worms fed with empty vector L4440 were used as a negative control, while *gtbp-1* RNAi was used as a positive control for knockdown validation where applicable. Protein bands were visualized using appropriate secondary antibodies and chemiluminescence detection. All experiments were performed in at least three independent biological replicates.

### Statistical analysis

Statistical analyses were performed using OriginPro 2017 (version 9.4.0). Details of statistical tests used for each experiment, including sample size, number of independent biological replicates, and definition of error bars, are provided in the corresponding figure legends. Unless otherwise stated, data are presented as mean ± SEM. Statistical significance was determined using tests appropriate for each dataset, as described in figure legends. All experiments were independently repeated at least three times unless otherwise indicated.

## Supporting information

S1 Fig GTBP-1 localization in the *C. elegans* germline under different temperature conditions. (A) Fluorescence microscopy of wild-type (control) and *gtbp-1(ust600)* mutant animals expressing GFP::HIS-58, grown at 20 ℃ or shifted to 25 ℃. This marker was used to visualize germline nuclear organization. Images are representative of at least three independent experiments, with ≥ 3 animals per condition. (B–D) Representative images of germlines from animals expressing GFP::GTBP-1 under different temperature conditions: control (20 ℃, B), chronic mild heat stress (25 ℃, C), and acute heat shock (37 ℃ for 30 min, D). Germline regions including the mitotic zone, pachytene, diplotene, and oocyte stages are indicated. Right panels show magnified views of GFP::GTBP-1 (green) and the nuclear envelope marker LMN-1::mCherry (magenta) in rachis, oocyte, and epidermal regions, with merged channels displayed. Images are representative of at least three independent experiments, with ≥ 3 animals per condition. (E) Full germline images showing GFP::GTBP-1 localization in animals exposed to chronic mild heat stress (25 ℃) or acute heat shock (37 ℃ for 30 min). These images include the complete rachis architecture to provide a tissue-scale view of GTBP-1 distribution across the entire germline. Images are representative of at least three independent experiments, with ≥ 3 animals per condition.
(TIF)

S2 Fig Functional domains of GTBP-1 regulate stress granule dynamics and reproductive fitness under temperature stress. (A) Quantification of stress granule enrichment in animals expressing full-length GFP::GTBP-1 or domain deletion mutants (ΔNTF2, ΔIDR, ΔRRM, ΔRGG) following acute heat stress (37 ℃ for 30 min). Granule enrichment was normalized to the surrounding cytoplasmic signal. Data are presented as mean ± SEM (n = 5 animals per condition). Each data point represents one animal. Statistical significance was determined using a two-tailed unpaired Student’s t-test. Representative results from at least three independent experiments are shown. (B) Representative confocal images showing GFP::GTBP-1 localization dynamics in the germline under acute heat shock (37 ℃ for 30 min) and recovery at 20 ℃ (30 min and 60 min). Full-length GTBP-1 and deletion mutants (ΔNTF2, ΔIDR, ΔRRM, ΔRGG) were analyzed. Images are representative of at least three independent experiments, with ≥ 3 animals per condition. (C) Brood size analysis of N2 and transgenic animals expressing full-length or domain-deleted GFP::GTBP-1 at 20 ℃. Each data point represents an individual worm (n = 15 animals per genotype). Box plots show median, interquartile range, and full data range. Statistical significance was determined using two-tailed unpaired Student’s t-test. (D) Brood size analysis of N2 and transgenic animals expressing full-length or domain-deleted GFP::GTBP-1 under chronic heat stress (25 ℃). Each data point represents an individual worm (n = 15 animals per genotype). Box plots show median, interquartile range, and full data range. Statistical significance was determined using two-tailed unpaired Student’s t-test.
(TIF)

S3 Fig Colocalization of GTBP-1 with stress granule markers in somatic and embryonic tissues under heat stress. (A) Representative confocal images of GFP::GTBP-1 (green) and PQN-59::mCherry (magenta) in the epidermis and early embryos (2-cell, 4-cell, and multicellular stages), grown at 20℃ or exposed to heat stress (37℃ for 30 min). Images are representative of at least three independent experiments, with ≥ 3 animals per condition. (B) Representative confocal images of GFP::GTBP-1 (green) and TIAR-1::mCherry (magenta) in the epidermis and early embryos (2-cell, 4-cell, and multicellular stages), grown at 20℃ or exposed to heat stress (37℃ for 30 min). Images are representative of at least three independent experiments, with ≥ 3 animals per condition.
(TIF)

S4 Fig Brood size of *gtbp-1(ust600)* following RNAi knockdown at 25 ℃. Brood size analysis of *gtbp-1* mutants at 25 ℃ following RNAi knockdown of candidate genes (n = 15 animals per genotype). Box plots show median, 25–75% percentiles, and minimum/maximum values. Statistical significance was determined using two-tailed unpaired Student’s t-test. Groups with complete infertility were not subjected to statistical analysis.
(TIF)

S5 Fig Heat stress promotes colocalization of GTBP-1 with the P-body component CGH-1 in the germline and early embryos. (A) Representative confocal images of germlines co-expressing GFP::GTBP-1 (green) and mCherry::CGH-1 (magenta) under control conditions (20 ℃) or after acute heat stress (37 ℃ for 30 min). Major germline regions, including the mitotic zone, pachytene, diplotene, and oocytes, are indicated. Higher-magnification views of the rachis, oocytes, and epidermis are shown on the right. Images are representative of at least three independent experiments, with ≥ 3 animals per condition. (B) Representative confocal images of 2-cell, 4-cell, and multicellular embryos co-expressing GFP::GTBP-1 (green) and mCherry::CGH-1 (magenta) under control conditions (20 ℃) or following acute heat stress (37 ℃ for 30 min). Boxed regions are shown at higher magnification to highlight GTBP-1/CGH-1-positive granules. Line-scan analyses of the indicated granules are shown on the right. Images are representative of at least three independent experiments, with ≥ 3 animals per condition.
(TIF)

S6 Fig Effects of candidate gene knockdown on GFP::GTBP-1 protein levels. Representative Western blot analysis of GFP::GTBP-1::FLAG protein levels following RNAi treatment with L4440, *gtbp-1*, *pqn-59*, *tiar-1*, *smo-1*, *rsks-1*, *laf-1*, *edc-3*, *cgh-1*, *or ifet-1*. GFP::GTBP-1::FLAG was detected using an anti-FLAG antibody. β-actin was used as a loading control. Molecular weights are indicated on the right. Representative Western blot from at least three independent experiments.
(TIF)

S1 Table *C. elegans* strains used in this study. (XLSX)

S2 Table Sequences of the sgRNAs. (XLSX)

S3 Table Sequences of repair plasmids primers. (XLSX)

S4 Table List of genes used in the RNAi screening. (XLSX)

S1 Data Source data underlying all graphs and quantifications presented in this study. (XLSX)

S1 Raw Gel. Uncropped and minimally processed images underlying the western blot figures. (PDF)
