# Supplementary material for: Heat stress promotes GTBP-1 association with perinuclear P-bodies in the C. elegans germline
Source: PLoS Genet. 2026 Oct 7;22(10):e1012336. doi: 10.1371/journal.pgen.1012336 (PMC13671640; doi:10.1371/journal.pgen.1012336)
Supplement: Supplementary file 1 [file pgen.1012336.s012.pdf]

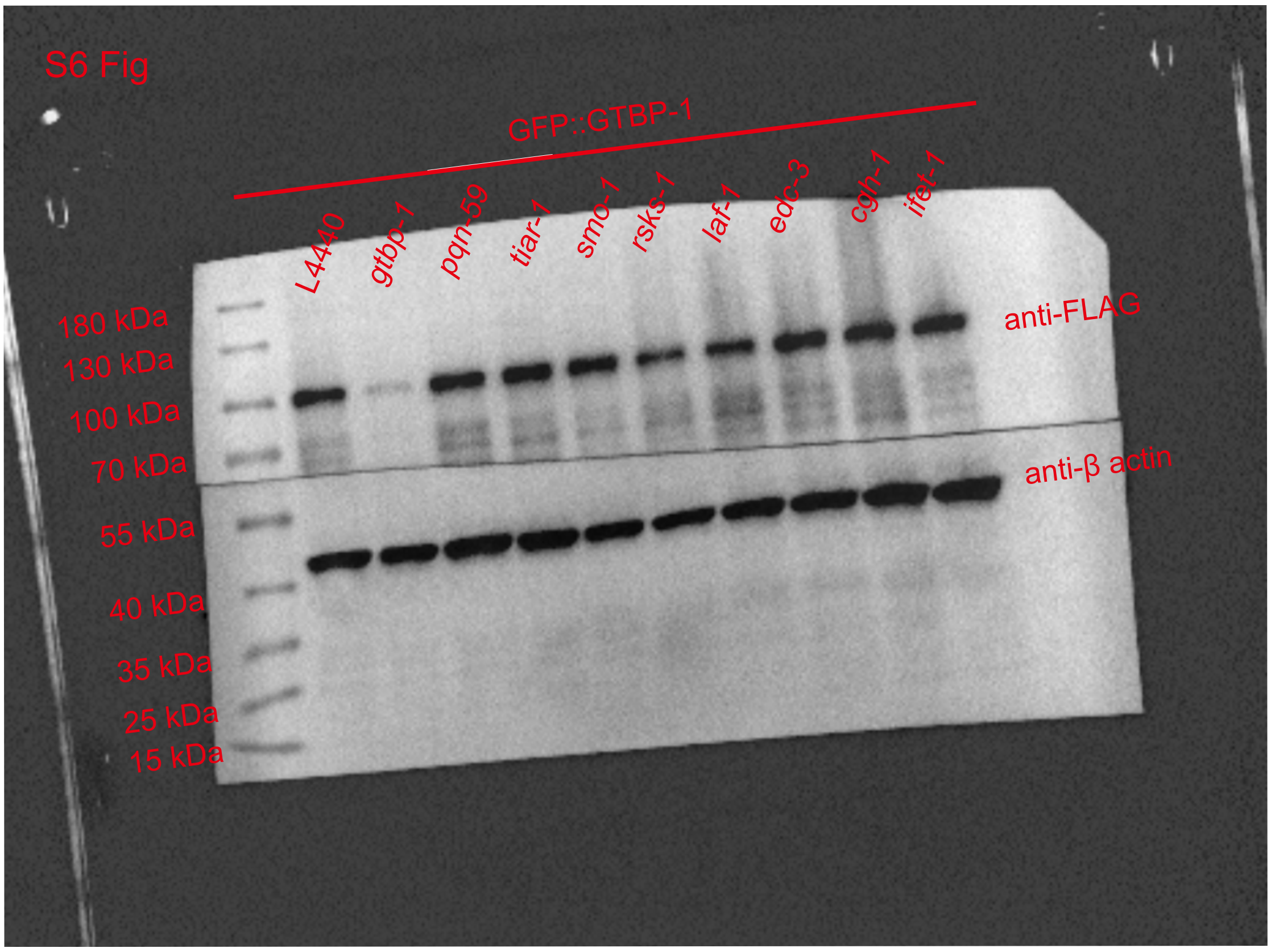

S6 Fig
GFP::GTBP-1
L4440
gtbp-1
pqn-59
tiar-1
smo-1
rsks-1
laf-1
edc-3
cgh-1
ifet-1
180 kDa
130 kDa
100 kDa
70 kDa
55 kDa
40 kDa
35 kDa
25 kDa
15 kDa
anti-FLAG
anti-β actin
